# Consumption of traditional alcoholic beverages in children from a rural village in Northern Peru, 2017

**DOI:** 10.12688/f1000research.12039.2

**Published:** 2018-03-21

**Authors:** Juan M. Ramírez-Ubillus, Martín A. Vilela-Estrada, Shirley A. Herrera-Arce, Estefany Mejía-Morales, Christian R. Mejia

**Affiliations:** 1 Escuela de Medicina Humana, Universidad Privada Antenor Orrego, Trujillo, 13007, Peru; 2 Escuela de Medicina Humana, Universidad Continental, Huancayo, 12000, Peru; 3 Escuela de Postgrado, Universidad Privada Antenor Orrego, Trujillo, 13007, Peru

**Keywords:** Alcohol, Children, Health Problems, Peru

## Abstract

**Introduction: **Alcoholic beverages have a proven impact on neuronal development and other areas of the body, primarily the heart, kidneys and liver, which is why their consumption in children is prohibited. However, there are traditional drinks that have alcohol content (Chicha de Jora-Clarito); artisanal drinks of traditional origin with alcoholic content in Peru. The aim of this study was to characterize the consumption of traditional alcoholic beverages in children of a rural village in Northern Peru.

**Methods: **This study was an analytical cross-sectional study. Mothers were recruited by census sampling and reported the consumption by their children of two traditional drinks with alcoholic content: Chicha de Jora (Ch) and Clarito (Cl), which are derived from the fermentation of maize. The frequency of consumption, accessibility and perception of consumption risk were described.

**Results**: Data were collected about 300 children, 61% (183) of whom consumed Ch. and 31% (92) of whom consumed Ch and Cl. Regarding drink accessibility, the majority of mothers said that these drinks were cheap (Ch: 69.0% and Cl: 60.7%). Additionally, the vast majority of families sometimes consumed or always consumed such beverages (Ch: 81.3% and CI: 65.7%). One in three mothers perceived Ch and Cl as being nutritious and helping their children grow. 25% of mothers perceived that there was no risk to their children from the consumption of the beverages, whereas >60% said that there could be a risk due to the beverages’ alcohol content.

**Conclusions**: Our study found that traditional beverages containing alcohol are consumed frequently by children in a village in Northern Peru. Mothers provide accessibility to the beverages and perceive the risk the drinks have, which will more accurately evaluate this risk. We advise that future studies concerning the intervention of these attitudes are performed, for a better future and development of children.

## Introduction

Alcoholic beverages, which are traditionally derived from the fermentation of sugars and yeast ^1^, currently have a large socio-economic impact. The World Health Organization states that 3.3 million deaths are caused every year worldwide by the harmful use of alcohol ^2^. It is well known that these types of drinks cause a series of physiological problems (renal, digestive, hepatic, etc.) ^3, 4^, as well as behavioral problems, which include maladaptation to the family and social environment, and, in extreme situations, could lead to suicide ^5^.

According to worldwide data, alcohol use has 5.1% comorbidity (high blood pressure, cirrhosis, renal disease, etc.) in the age group between 20–39 years ^6^. However, some countries, such as Colombia and Argentina, have reported onset at an earlier age ^7^. In Peru, there is almost no information on this subject (information that is provided is mostly provided by local institutions); however reports show that the median age when alcohol consumption begins is 13 years, while in locations where children have greater access to alcoholic beverages, consumption starts at 10 years ^8^.

Chicha de Jora (Ch) and Clarito (Cl) are drinks derived from the fermentation of maize that have been consumed since Pre-Hispanic times throughout the northern coast of Peru. The Incas, among the types of corn that they cultivated considered the germinated corn (Jora) as a sacred drink; giving two derivatives of alcoholic content (Chicha de Jora and Clarito). This tradition has been passed from generation to generation until today. Currently, the elaboration of this millenary drink in Peru (especially in the North Coast) is done by hand, not having a formal regulation by the industry ^9^; thus reaching about 28.7% of unregistered alcohol registered by the World Health Organization (WHO) ^10^, since consumers despite having between 10 to 12 degrees of alcohol, they consider this as a traditional drink ^10^.

Consumption is high due to their low production cost, ease of access, and tradition ^11^. These factors can create a problem if such drinks are consumed by children and teenagers. The objective of this study was to characterize the consumption of these traditional alcoholic beverages in children of a rural village in Northern Peru.

## Methods

### Design and study population

A cross-analytical cross-sectional study was carried out between February and May 2017, in which the mothers and/or guardians of the Northern Peruvian settlement of "La Piedra", where 308 children under the age of 15 reside, were surveyed. Household visits were completed for the purposes of the study. Thanks to the information provided by the governor, the surveys were carried out in each of the homes of the mothers and/or guardians using census sampling. A sample size was calculated for a descriptive study, for the local population of children, with a statistical power of 99%, a 95% confidence level and a maximum prevalence of 50%. A minimum sample of 300 children was obtained; this was captured non-randomly.

All mothers residing in the populated center (small town) during the interview were included. Mothers who did not wish to participate in the study, as well as those mothers who responded inadequately to our survey were excluded. After reading through the informed consent and agreeing to participate the mothers were enrolled in the study. Those who did not respond adequately to the survey (unanswered questions and/or incomplete answers) were excluded. Rate of rejection = 2.5%, thus achieving a total of 300 surveys applied, obtained from the interview of 103 mothers or guardians (in some cases the mothers or guardians had more than one child).

### Survey design

For the present study, a survey was carried out, which was previously validated by a pilot study in a sample of 50 individuals, where a Cronbach's alpha of 0.781 was obtained. The previous pilot study was not published, the results were only for the evaluation of the survey. The survey had minor modifications after the pilot study. These were used to specify the details of consumption, access and even the consequences of the consumption of alcoholic beverages. The final survey had two main sections ( Supplementary File 1):

*Socio-demographic data:* Basic data was provided, such as the child’s age, weight, height and school grade, and in addition the number of household members and household income.

*Characteristics of drinking habits in liquids/beverages:* These characteristics were evaluated through closed questions, in which inquiries were made about the daily consumption of different drinks, primarily the consumption of beverages containing alcohol (Ch and Cl). The following information was obtained: the frequency of consumption (1 day a week; 2 days a week; Every day; No consumption), the accessibility of beverages (Very low cost - less than S/.1 Sun/Bottle; Low cost - less than S/.5 Soles/Bottle), whether or not consumed by the person who responded to the survey and by the whole family, and if the consumption of the beverages was perceived as harmful or nutritional for the child's health. Finally, other exploratory variables were captured, such as the consumption of other types of beverages (aerated beverages, pure water, milk, lemonade, Chicha Morada, etc. - all of them without alcohol content); and a section where the child's socio-academic problems were assessed was included. These exploratory variables are not discussed in the present study.

All surveys were anonymous and were conducted by a researcher belonging to the study. The approximate duration of the survey was 20 minutes. At all times the assigned researcher was properly trained to be able to solve doubts about any of the questions.

### Data analysis

For the data analysis, a double digitizing system (data processed by two researchers separately, and then checked for errors manually) was performed, for a better control of the data collected. Surveys were entered in the Microsoft Excel program (version 2015), then proceeded to make a first filter for checking the data. Following this, the data were processed in Stata 11.1 (StataCorp LP, College Station, TX, USA).

For descriptive statistics, we worked with frequencies/percentages for categorical variables, and medians and interquartile ranges for the quantitative variables. The chi-square statistical test was applied for the association of the consumption of the drinks versus the perception that the consumption of the drinks could be bad for children. P<0.05 was considered statistically significant.

### Ethical statement

Permission and support was provided by local authorities (governor, health center doctor and school director). Since children were the target of this study, all precautions were taken to ensure anonymity and respect for ethical precepts. The study was approved by the Ethics Committee of the San Bartolomé National Hospital, endorsed by the National Health Institute (NIH; approved March 5, 2016; Office No. 422). This committee was chosen since there is no committee that monitors the approval of the NIH where the study was conducted. This committee also approved the pilot study. The ethical standards on human experimentation of the Declaration of Helsinki of 1975 were taken into account. The results will be given to the sanitary authorities of the region, so that they can learn about this reality and put forward strategies of help. The study was carried out under the permission of the mothers/guardians, who gave written informed consent.

## Results

Data were collected about 300 children, 51.3% (154) were girls, and the median age was 9 years (interquartile range: 5–12 years). 15.8% (41) studied at an initial level, 53.5% (139) studied in a primary school and 30.7% (80) studied in secondary school. 61.0% (183) and 30.7% (92) consumed Ch and Cl, respectively ( Table 1).

**Table 1.** Consumption of traditional alcoholic beverages in children from a rural village in Northern Peru (n=300). Quantitative values are presented in median (interquartile range).

Beverage	Child consumption, n (%)	Consumption frequency, per week	Consumption initiated, years
Chicha de Jora	183 (61.0)	3 (1-7)	3 (2-5)
Clarito	92 (30.7)	3 (1-7)	4 (2-5)

Most of the mothers reported that they consumed Ch (84.7%) and Cl (62.7%) when they were children, and the majority also consume the drinks now (Ch: 74.0% and Cl: 47.7%). Regarding accessibility of the beverages, the majority of mothers said that these drinks were cheap (Ch: 69.0% and Cl: 60.7%), and the vast majority of families sometimes consumed or always consumed such beverages (Ch: 81.3% and Cl: 65.7%) ( Table 2).

**Table 2.** Accessibility of traditional alcoholic beverages to children from a rural village in Northern Peru (n=300).

	Chicha de jora, n (%)	Clarito, n (%)
**Mother’s consumption**		
Consumed as child	254 (84.7)	188 (62.7)
Consume currently	222 (74.0)	143 (47.7)
**Accessibility**		
Thought to be cheap	207 (69.0)	182 (60.7)
Family consumption		
* Never*	56 (18.7)	103 (34.3)
* Sometimes*	175 (58.3)	159 (53.0)
* Always*	69 (23.0)	38 (12.7)

**Figure 1.**
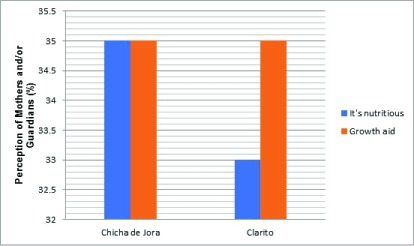
Mothers’ perceptions of nutrition and aid for growth provided by traditional alcoholic drinks consumed by children.

35% of mothers perceived that Ch is nutritious and helps growth, while 33% and 35% of mothers perceived that Cl is nutritious and helps growth, respectively ( Figure 1). 25% of mothers perceived that there was no risk for their child to consume the beverages. However, >60% said that there could be a risk due to the alcohol contained in the drinks ( Table 3).

**Figure 2.**
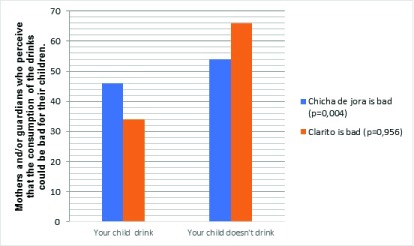
Percentage of consumption of alcoholic beverages among mothers who perceived that drinks could be bad for their children.

Figure 2 shows that although women perceive consumption of beverages as bad for their children, 46% and 34% still gave their children Ch and Cl, respectively.

**Table 3.** Perception of risk of traditional alcoholic beverages for children of a rural village in Northern Peru (n=300).

	Chicha de jora, n (%)	Clarito, n (%)
**Health benefits**		
Not dangerous	59 (25.8)	56 (24.8)
Dangerous	Not dangerous (0)	Not dangerous (0)
**Reasons for danger**		
Alcohol content	145 (63.3)	150 (66.4)
Religion forbids it	6 (2.6)	0
Does not help knowledge	3 (1.3)	3 (1.3)
Decreases intelligence	3 (1.3)	3 (1.3)
Other	13 (5.6.)	14 (6.1)

Raw data from the responses of mothers/guardians concerning their children’s consumption of traditional alcoholic beverages (n=300 children) Click here for additional data file. Copyright: © 2018 Ramírez-Ubillus JM et al. 2018 Data associated with the article are available under the terms of the Creative Commons Zero "No rights reserved" data waiver (CC0 1.0 Public domain dedication).

## Discussion

The consumption of alcohol in children is still a very important problem, as evidenced in this study, where out of 300 children surveyed, 183 and 92 children consumed Chicha de Jora (Ch) and Clarito (Cl), respectively, every week. These results of consumption are greater than in different studies from different countries. For example, in Brazil, only 12.8% of children consumed any type of alcoholic beverage before age 10 ^12^; in the Province of Buenos Aires, 55.4% of adolescents between the ages of 11 and 14 consume alcohol ^13^; while a study in Colombia, with children at the mean age of 14.4 years, concluded that the pattern of alcohol abuse measured by the CAGE scale was 14.6% ^14^.

The consumption of these traditional beverages also occurred during the mothers' childhood, with a majority stating that they had consumed both drinks. Many of the mothers expressed that they still consume them. A report of a population study in Chile, of 408 alcoholic respondents, reported that 27.2% lived with children in the house and in 46.3% of cases the drinker was either the father or the mother ^15^. Another report in Angola showed that 56% of mothers of 319 children had regular alcohol habits. Our study showed that this percentage was higher at 84.7% of mothers who consume Ch and 62.7% who consume Cl ^16^. Also in Brazil, Argentina, Colombia, Chile, and Mexico, it was reported that occasional consumption of alcohol is associated with family context, influence of friends, antisocial behavior, and skills and experiences already acquired in childhood, which could be circumstances that encourage the consumption of alcohol in children ^12– 14, 17, 18^.

The consumption of alcohol in younger populations has risen in recent years in Peru, which has the potential to cause harm and create addictive behavior ^18^. In our population, the acquisition of Ch (69.0%) and Cl (60.7%) was considered economical-average cost: 1 to 5 Soles / Bottle (0.80 Euros)- because of their low cost of production; therefore making them more accessible and frequently consumed. One in every three mothers perceived that the Ch and Cl are nutritious and help the growth of their children, and this is a perception that could lead them to giving these drinks to their children. A study from Spain reported that fathers and mothers do not consider their children's alcohol consumption to be a problem ^19^, thus increasing their early intake without restriction.

In the present study, most mothers knew about the risk of alcohol consumption by children. However, it was observed that the consumption in most of their children remained high. Studies carried out in Spain and Cuba indicate that the family can be a protection, but also a risk factor. In both cases, the maternal figure tends to have a positive influence on the child, which differs from what was found in the present study ^19, 20^. We can infer that this is mainly due to a socio-cultural characteristic where the community (and especially the mothers) view the consumption of these traditional alcoholic beverages as normal.

The study had the limitation of selection bias, since it was completed in a sample that does not represent the total population of Peru. Likewise, since this is a preliminary study, its non-quantitative nature also counts as a limitation. However, this study used census-type sampling in a population that had not been previously reported; therefore, these results can be taken as preliminary. In particular, these findings can be used to alert the responsible authorities, so that detection and support measures can be implemented, so that families in this village and similar locations with similar consumption conditions can receive the necessary support.

## Conclusions

According to the present study, it is concluded that children consume traditional alcoholic beverages and that their mothers provide access. Although mothers perceive the risk that these drinks have, they still give them to their children. Finally, there could be a danger to health, however, further studies would be necessary in a quantitative manner, which would more accurately assess this risk.

## Data availability

The data referenced by this article are under copyright with the following copyright statement: Copyright: © 2018 Ramírez-Ubillus JM et al.

Data associated with the article are available under the terms of the Creative Commons Zero "No rights reserved" data waiver (CC0 1.0 Public domain dedication). 

**Dataset 1:** Raw data from the responses of mothers/guardians concerning their children’s consumption of traditional alcoholic beverages (n=300 children). doi, 10.5256/f1000research.12039.d170158 ^21^

## Supplementary material

**Supplementary File 1: Survey for mothers/guardians relating to the consumption of traditional alcoholic beverages in their children.** This survey is provided in Spanish and English.
