# Supplementary material for: Consumption of traditional alcoholic beverages in children from a rural village in Northern Peru, 2017
Source: F1000Res. 2018 Mar 21;6:1270. Originally published 2017 Jul 28. [Version 2] doi: 10.12688/f1000research.12039.2 (PMC6259489; doi:10.12688/f1000research.12039.2)
Supplement: Supplementary file 2 [file f1000research-6-15625-s0001.tgz › b725593c-8812-4e0b-bdb4-d26c4b17a82a.docx]

**FICHA DE RECOLECCIÓN DE DATOS**

**DATOS DEL NIÑO (MENOR DE EDAD)** Fecha de recolección: ___/___/___

1. Sexo: Masculino ( ) Femenino ( )
2. Edad: ____ años
3. Peso: _________Kg.
4. Talla: ________cms.
5. ¿Cuántas personas integran la familia (que viven en su casa)? ____________
6. Sumando todos los ingresos económicos, cuánto entra de dinero al mes: _____________
7. ¿En qué etapa académica está? Inicial ( ) Primaria ( ) Secundaria ( )
8. ¿Qué grado del colegio cursa? 1 ( ) 2 ( ) 3 ( ) 4 ( ) 5 ( ) 6 ( )
9. Por favor responder acerca de los líquidos/bebidas que toma el niño en una semana promedio:

| **Bebida** | ¿La consume su hijo? | ¿Cuántas veces  a la semana? | ¿Hace  cuántos  años? |
| --- | --- | --- | --- |
| Agua pura | No ( ) Si ( ) |  |  |
| Gaseosa | No ( ) Si ( ) |  |  |
| Leche | No ( ) Si ( ) |  |  |
| Limonada o chicha morada | No ( ) Si ( ) |  |  |
| Chicha de jora | No ( ) Si ( ) |  |  |
| Clarito | No ( ) Si ( ) |  |  |
| Otras bebidas | No ( ) Si ( ) |  |  |

Ahora le pedimos que responda algunas preguntas de las características de estas bebidas:

| **Bebida** | ¿Es barata o  cuesta poco? | ¿Yo la tomé  de niña(o)? | ¿Toda nuestra familia  la consume? |
| --- | --- | --- | --- |
| Agua pura | No ( ) Si ( ) | No ( ) Si ( ) | Siempre ( ) A veces ( )  Nunca ( ) |
| Gaseosa | No ( ) Si ( ) | No ( ) Si ( ) | Siempre ( ) A veces ( )  Nunca ( ) |
| Leche | No ( ) Si ( ) | No ( ) Si ( ) | Siempre ( ) A veces ( )  Nunca ( ) |
| Limonada/chicha morada | No ( ) Si ( ) | No ( ) Si ( ) | Siempre ( )  A veces ( )Nunca ( ) |
| Chicha de jora | No ( ) Si ( ) | No ( ) Si ( ) | Siempre ( ) A veces ( )  Nunca ( ) |
| Clarito | No ( ) Si ( ) | No ( ) Si ( ) | Siempre ( ) A veces ( )  Nunca ( ) |

A continuación, le pedimos que valore cada una de las bebidas

| **Bebida** | ¿Es nutritiva? | ¿Ayuda en el  crecimiento? | ¿Podría ser malo su  consumo? Si su  respuesta es SI,  decir el Por qué |
| --- | --- | --- | --- |
| Agua pura | No ( ) Si ( ) | No ( ) Si ( ) | No ( ) Si ( ), Xq: |
| Gaseosa | No ( ) Si ( ) | No ( ) Si ( ) | No ( ) Si ( ), Xq: |
| Leche | No ( ) Si ( ) | No ( ) Si ( ) | No ( ) Si ( ), Xq: |
| Limonada/chicha morada | No ( ) Si ( ) | No ( ) Si ( ) | No ( ) Si ( ), Xq: |
| Chicha de jora | No ( ) Si ( ) | No ( ) Si ( ) | No ( ) Si ( ), Xq: |
| Clarito | No ( ) Si ( ) | No ( ) Si ( ) | No ( ) Si ( ), Xq: |

Por último, queremos que nos mencione acerca de algunos problemas que pudo

haber tenido su hijo:

| **Problemas** | **¿Su hijo(a) lo**  **ha tenido?** | **¿Cuántas veces en toda su vida? (Aprox)** | **¿Hace cuantos meses fue la última vez?** |
| --- | --- | --- | --- |
| Peleas entre hermanos/familia | No ( ) Si ( ) |  |  |
| Peleas en el colegio | No ( ) Si ( ) |  |  |
| Mala conducta en casa | No ( ) Si ( ) |  |  |
| Bajas notas en el colegio | No ( ) Si ( ) |  |  |
| Repetir un año | No ( ) Si ( ) |  |  |
| Mala conducta en el colegio | No ( ) Si ( ) |  |  |
| Problemas de salud que haya generado que falte al colegio | No ( ) Si ( ) |  |  |

**DATA COLLECTION DATA SHEET**

**CHILD'S DATA (MINOR)** Date of collection: ___ / ___ / ___

1. Gender: Male () Female ()

2. Age: ____ years

3. Weight: _________Kg.

4. Size: ________cms.

5. How many people are in the family (living in your home)? ____________

6. How much is the monthly income?: _____________

7. At what academic stage is it? Initial () Primary () Secondary ()

8. What grade of school do you attend? 1 () 2 () 3 () 4 () 5 () 6 ()

9. Please answer about the liquids / drinks the child takes in an average week:

| **Drink** | **Does your child**  **consume it?** | **How often**  **a week?** | **For**  **how many**  **years?** |
| --- | --- | --- | --- |
| Pure water | No( ) Yes ( ) |  |  |
| Soda | No ( ) Yes ( ) |  |  |
| Milk | No ( )Yes ( ) |  |  |
| Lemonade /Chicha Morada da | No( ) Yes ( ) |  |  |
| Chicha de jora | No ( )Yes ( ) **(1)** | **(2)** | **(3)** |
| Clarito | No ( ) Yes ( ) **(4)** | **(5)** | **(6)** |
| Other Drinks | No ( ) Yes ( ) |  |  |

Now we ask you to answer some questions about the characteristics of these drinks:

| **Drink** | **Is it cheap/does it cost**  **little?** | **Do you drink it**  **the same as**  **the child?** | **Does the whole family**  **consume it?** |
| --- | --- | --- | --- |
| Pure water | No ( ) Yes ( ) | No ( ) Yes ( ) | Always ( ) Sometimes ( )  Never ( ) |
| Soda | No ( ) Yes ( ) | No ( ) Yes ( ) | Always ( ) Sometimes ( )  Never ( ) |
| Milk | No ( ) Yes ( ) | No ( ) Yes ( ) | Always ( ) Sometimes ( )  Never ( ) |
| Lemonade /  chicha morada | No ( ) Yes ( ) | No ( ) Yes ( ) | Always ( ) Sometimes ( )  Never ( ) |
| Chicha de jora **(7)** | No ( ) Yes ( ) **(8)** | No ( ) Yes ( ) **(9)** | Always ( ) Sometimes ( ) **(10)**  Never ( ) |
| Clarito **(11)** | No ( ) Yes ( ) **(12)** | No ( ) Yes ( ) **(13)** | Always ( ) Sometimes ( ) **(14)**  Never ( ) |

Then, we ask you to rate each of the drinks

| **Drink** | **Is it nutritious?** | **Does it help**  **growth?** | **Could it be bad for you?**  **If your answer is YES,**  **state why** |
| --- | --- | --- | --- |
| Pure water | No ( ) Yes ( ) | No ( ) Yes ( ) | No ( ) Yes ( ), Why: |
| Soda | No ( ) Yes ( ) | No ( ) Yes ( ) | No ( ) Yes ( ), Why: |
| Milk | No ( ) Yes ( ) | No ( ) Yes ( ) | No ( ) Yes ( ), Why: |
| Lemonade /  chicha morada | No ( ) Yes ( ) | No ( ) Yes ( ) | No ( ) Yes ( ), Why: |
| Chicha de jora | No ( ) Yes ( ) **(15)** | No ( ) Yes ( ) **(16)** | No ( ) Yes ( ), Why: **(17) (21)** |
| Clarito | No ( ) Yes ( ) **(18)** | No ( ) Yes ( ) **(19)** | No ( ) Yes ( ), Why: **(20) (22)** |

Finally, we want you to tell us about some problems your child may have had:

| **Problems** | **Has your child**  **been involved in?** | **How many times in your life? (Approximately)** | **How many months ago was the last time?** |
| --- | --- | --- | --- |
| Fights between siblings / family | No ( ) Yes ( ) |  |  |
| Fights at school | No ( ) Yes ( ) |  |  |
| Bad behavior at home | No ( ) Yes ( ) |  |  |
| Low notes at school | No ( ) Yes ( ) |  |  |
| Repeat one year | No ( ) Yes ( ) |  |  |
| Bad behavior in school | No ( ) Yes ( ) |  |  |
| Health problems caused by missing school | No ( ) Yes ( ) |  |  |

**Coding:**

| **ID** | **Question** | **Coding of responses** |
| --- | --- | --- |
| **1** | Chicha_Jora (Ch) | No = 0; Yes= 1 |
| **2** | Ch._Week | Answer in numbers |
| **3** | Ch.Years | Answer in numbers |
| **4** | Clarito (Cl) | No = 0; Yes= 1 |
| **5** | Cl_Weeks | Answer in numbers |
| **6** | Cl_Years | Answer in numbers |
| **7** | Ch_consumption | No = 0; Yes= 1 |
| **8** | Ch_cheap | No = 0; Yes= 1 |
| **9** | Ch_drink | No = 0; Yes= 1 |
| **10** | Chi_fam_cons | Always= 0 Sometimes=1  Never =2 |
| **11** | Cl_cons | No = 0; Yes= 1 |
| **12** | Cl_cheap | No = 0; Yes= 1 |
| **13** | Cl_drink | No = 0; Yes= 1 |
| **14** | Cl_fam_cons | Always= 0 Sometimes=1  Never =2 |
| **15** | Ch_nutri | No = 0; Yes= 1 |
| **16** | Ch_crec | No = 0; Yes= 1 |
| **17** | Ch_bad | No = 0 Yes/ Why: |
| **18** | Cl_nutri | No = 0; Yes= 1 |
| **19** | Cl_crec | No = 0; Yes= 1 |
| **20** | Cl_bad | No = 0 Yes/ Why: |
| **21** | Ch_Bad | No = 0; Yes= 1 |
| **22** | Cl_bad | No = 0; Yes= 1 |

**Note:** The assigned codes belong to the questions used to compile the data table
