# Supplementary material for: Consumption of traditional alcoholic beverages in children from a rural village in Northern Peru, 2017
Source: F1000Res. 2018 Mar 21;6:1270. Originally published 2017 Jul 28. [Version 2] doi: 10.12688/f1000research.12039.2 (PMC6259489; doi:10.12688/f1000research.12039.2)
Supplement: Peer review history

# Peer review: Referee response for version 2

**DOI:** 10.5256/f1000research.15625.r39793

**Published:** 2018-11-26

**Authors:** Maria Neufeld

**Affiliations:** 1 Dresden University of Technology, Dresden, Germany

The study provides very interesting and valuable insights into a topic, which is still very under-researched. However, I would recommend including some changes to improve clarity and flow of the manuscript and also to help settle the conclusion, as for now, it is not entirely clear where exactly the “health danger” is coming from and what exactly is problematic about the observed behaviours. 

1. Please think of introducing further keywords to your article to make it more accessible/easier to find for an international readership. I would advise on including the key words “unrecorded alcohol” and “alcohol drinking”, for instance.
2. In the abstract please clarify what exactly you mean with “the majority of mothers said that these drinks were cheap (Ch: 69.0% and Cl. 60.7%).” It took me a while to understand what exactly you mean with the numbers and I finally got it with the help of Table 2, which indicates that these percentages relate to the question whether the mothers/legal guardians consider these drinks to be cheap. So I would suggest to rephrase the abstract a bit: “the majority of mothers said that these drinks were cheap (69% considered Ch. to be cheao and 60.7% considere Cl.to be cheap). The explanation of what was meant with cheap should appear already in the introduction or the methods section and not in the discussion section.
3. I fear that I do not fully understand what you mean in the discussion when saying “In our population, the acquisition of Ch (69.0%) and Cl (60.7%) was considered economical-average cost: 1 to 5 Soles / Bottle (0.80 Euros)- because of their low cost of production; therefore making them more accessible and frequently consumed.” --> so what do you say here and how does this, again, relate to the perception of these beverages as being cheap?
4. Please clarify whether there is a difference between the two traditional drinks Ch and Cl in terms of alcohol content or any other property that might be of interest/importance. For now the manuscript states that both beverages are derived from fermentation of maize, have an alcohol content of about 10-12%  and that Cl is slightly cheaper (?). Is there anything else the readership should know? Please add some ideas to the discussion section why you think the consumption of Cl was less frequently reported than consumption of Ch and why Cl. was perceived as less nutritious. The international readership is not familiar with these two beverages and it would be good to get an idea of their differences, after having read the text.
5. It would be good to have some clarity on certain terms.
  - For instance, please use the term “unrecorded alcohol” instead of “unregistered”, especially when referring to the WHO definition.
  - Also please use the term “affordability”, when describing and discussion the prices of the beverages. Accessibility, at least in my view, rather relates to the question of physical access of the children to alcohol, specifically the questions whether mothers/guardians were given the alcoholic beverages to the children or not. So please separate the two terms for better clarity.
  - Please be very clear that you always refer to mothers AND guardians in the manuscript, unless you talk about consumption of the different beverages in the family, which is a different indicator according to your questionnaire. You might want to introduce a footnote or an abbreviation at the very beginning. For now, the terms “mothers” and “mothers and guardians” are used inconsistently throughout the text.
6. Please double-check what was already raised by another reviewer: --> Table 3: check values for line “dangerous”. Is this logical that both are “0”?” The table is a bit confusing the way it is, maybe you could rearrange for better readability.
7. When citing the Global Status Report on Alcohol and Health of the WHO, please refer to the newest one from September 2018 and please also update the numbers you cite from the report ^1^.
8. Also, I do not quite understand why you cite some indicators from the GSRAH on comorbidities in the age group of 20-39 years old. How does this relate to the subject of your study? You look at a much much younger cohort, so citing evidence from the same age group would be more appropriate here. Moreover, I do not understand why you cite comorbidities here. The way I see it, the GSRAH provides much more important indicators: for instance the percentage of total deaths attributable to alcohol, by age group for the WHO region of the Americas (Fig 4.17 of the report). If there’s something that should be cited from the GSRAH, then definitely this, highlighting how alcohol is impacting on mortality relatively early in life. Also, the report provides a country profile for Peru, which could be also helpful for the authors, e.g. for the estimate of the share of unrecorded alcohol consumed in Peru (however, there’s nothing specific on young drinkers in it besides prevalence of heavy episodic drinking in the age group 15-19, which is not really helpful for this contribution).
9. Please be clear with what you mean “It is well known that these types of drinks cause a series of physiological problems” (Introduction, 3 ^rd^ sentence). The way it is put, it sounds very ambiguous, allowing for the conclusion that traditional alcoholic drinks cause problems, which would not have occurred if these were industrial alcoholic beverages. The authors should be very clear on where they stand here. For now, the evidence (Rehm et al., 2013) shows that the most harmful ingredient of unrecorded alcohol is ethanol and that traditional/homemade alcoholic beverages are generally of the same quality as industrially manufactured alcoholic beverages and that their harm stems rather from the cheap price and broader availability and according drinking patterns rather than chemical properties.
10. When discussing alcohol consumption of young people (in the introduction and the discussion) in the introduction, please refer either in the introduction or the discussion to some literature on the early age at onset of alcohol use and subsequent risk of developing an alcohol use disorder. I’m not an expert in the field, but these are the studies that come to my mind immediately:
  - Hingson, R. W., Heeren, T., & Winter, M. R. (2006) ^2^
  - Grant, B. F., & Dawson, D. A. (1997) ^3^
11. The following sentence of the introduction was very confusing to me:
  “Currently, the elaboration of this millenary drink in Peru (especially in the North Coast) is done by hand, not having a formal regulation by the industry; thus reaching about 28.7% of unregistered alcohol registered by the World Health Organization (WHO), since consumers despite having between 10 to 12 degrees of alcohol, they consider this as a traditional drink).”
  What is it the authors intend to say here? I do not quite understand how the different statements relate to each other. Just because something is a traditional alcoholic drink, it does not mean that it automatically belongs to the category unrecorded alcohol (again, please use the term “unrecorded” and not “unregistered” here). There are plentiful examples where the industry is producing alcoholic beverages that were/are traditionally homemade/handmade. Also please think twice about your statement of “formal regulation by the industry”. Is industry really the ones, who should be regulating this? What about the role of the government and public health in this? I would advise in being very careful in the way you argue here, as for now, the sentence suggests that the traditional beverages are somehow worse than industrially produced beverages or, at least, one can read those sentences like this.
  Also, please update the share of unrecorded alcohol in Peru. According to the newest WHO report, it is 19.1% for the latest available period (2016).
12. Please provide a bit more context and references to the claim: “Consumption is high due to their low production cost, ease of access, and tradition.” Unfortunately, I could not access the according reference for this sentence (11) to see to what source of information you refer here. Also, do you mean by “ease of access” physical availability of Ch. and Cl. and/or other factors, for instance the possibility to purchase these products when the local shops are closed at certain days/times? Further, you argue that “These factors can create a problem if such drinks are consumed by children and teenagers.” Please be more specific with what exactly you mean here. There are certain reasons why these drinks are consumed by children. Besides cheap price and higher availability (is it?) and the belief that these drinks are beneficial for the children’s growth, the legal minimum age for purchasing alcohol seems to another very important factor, which should be mentioned here explicitly.
13. The methods section should mention that this study was basically a household study and the limitations section should mention all the biases that naturally comes with assessing alcohol consumption in household surveys in general: selection bias (you did not ask children living in institutions, for instance), recall bias (mothers/guardians might forget how often they actually drink these beverages), social desirability bias and potential stigma of consumption (should be at least discussed whether this applies here or not) and so forth… Also, the methods and limitations section should explicitly mention and discuss the fact that children were actually not asked directly about their consumption and that the survey was done with mothers/legal guardians as proxies. It also should be mentioned/described how the interview situation exactly looked like and whether the children were present during the interview or not and how this could have influenced the results.
14. The methods section states that “all mothers residing in the populated center (small town) during the interview were included”, indicating that the sample was a census. What exactly does this sentence mean, however? Does it mean that mothers of the entire town were interviewed? Or of the city center only? I’m a bit confused by the choice of words here. If only mothers/legal guardians from the city center were included, please discuss what implications this might have in terms of representatively of the results.
15. Physiological indicators (weight and height of the children) were included, but not reported in the results section. Could the authors please kindly comment on that and decide whether they want to perform the analysis for these indicators or not. If these indicators are included, please report on whether these were collected through objective measuring or self-report by the mothers/guardians. If the latter is the case, please discuss the biases coming with this type of assessment.
16. Was there any kind of additional information available on the indicator/questions “Could it [the consumption of a certain drink] be bad for you?”? Does “the bad for you” refer to the mothers/guardian OR the child? If it refers to the mother/guardian, was there any kind of question that was asked for the child?
  I think it would be quite important to know how this “bad for you” is conceptualized by the authors and perceived by the interview partners. In table 3, the authors talk about “the perception of risk” of the mothers/guardians and then the binary variable of “dangerous”/“nor dangerous”. I wonder if it is justified to present this as risk perception and the outcomes as dangerous/not dangerous, if the original question asked very vaguely if consumption “is bad or not”. So far, I didn’t get the grasp of what the mothers/guardians were actually thinking about this concept. For instance, based on the information provided in table 3, the mothers/legal guardians seem to be aware of the fact that these drinks contain xy% of alcohol and that alcohol poses certain risks for the child’s development. However, my impression is that there is a certain discrepancy between the questionnaire and the ways the results are presented in terms of risk perception (e.g. that “bad for you” is something else than “dangerous”). Please report if there was already some clarity on that from the pilot, how the “bad for you” is related to risk perception. Maybe you even did some qualitative interviews in the pilot to understand how the risks are perceived by the mothers/legal guardians?
17. I think Figure 1 could be extended and include also the information of how many mothers/guardians thought that consumption of Ch. and Cl. is bad for them [again, based on whether this indicator was valid for the children or the mothers/guardians].
18. Please make sure that the titles of the tables/figures are consistent, e.g. the tables feature additional information on the sample size and study location and the figures do not.
19. Would be helpful, if Table 1 would feature some more information/broader definitions on “reason for danger”- e.g. what exactly does it mean “Does not heal knowledge”?
20. Figure 3 should report p values according to the F1000 article guidelines.
21. The second sentence in the discussion section states that “These results of consumption are greater than in different studies from different countries. For example,…”. Please make absolutely sure that you are comparing your results with the results of similar studies from other countries, i.e. with studies that have specifically measures consumption of unrecorded and traditional alcoholic beverages in children. I do not think that it is appropriate to compare your study with studies that were measuring consumption of recorded alcoholic beverages in children/youth. Basically, your results can be interpreted in a way that ¼ of the mothers/legal guardians did not perceive Ch. and Cl. as alcoholic beverages and therefore “bad for children”. So if asked if their children consume alcoholic products, they would have said “no”, although the opposite was the case. This simply means that you cannot compare your specific results to other studies if they were measuring something else and state that the children of your sample had a higher prevalence of alcohol consumption. However, this does not mean that you should omit comparisons with other studies from the Americas completely; it just means that one should compare apples with apples.
22. Please comment on the first reviewer’s remark: “Discussion, last paragraph: please include the non-quantitative nature as limitation. Considering your comment that “the consumption of these traditional alcoholic beverages” is seen as “normal”, is there at least some information how much of the beverages is consumed by the children?”
23. I would disagree with the claim that “since this is a preliminary study, its nonquantitative nature also counts as a limitation.” This IS a quantitative study, using a quantitate survey as instrument and featuring descriptive statistics. It does not provide risk-assessment and, as outlined by the first reviewer, but saying that this as a not quantitative study is also not justified. So I would advise on naming the lack of data on quantitative intake as the actual limitation/feature of the preliminary nature of the study.
24. I am also a bit confused by the sentence “However, this study used census-type sampling in a population that had not been previously reported; therefore, these results can be taken as preliminary.” I do not see the relation between census type sampling and the preliminary nature of the results. Of course, the study does not represent the entire population of Peru, but it fully represents the population of children living in household in this specific village. Therefore, it is representative of this specific village and might be representative of further villages of Norther Peru with similar characteristics. I do not see why these results should preliminary if talking only about this context.
25. Could you maybe be a bit more specific when saying “These findings can be used to alert the responsible authorities”. If I understand your finding correctly, the lack of awareness about the risks of alcohol for young people is one of the biggest issues in these communities. So I think it would be good to name this and some other issues specifically, as well as to write just one or two sentences what the authors think could be done/what kind of action is needed. I think that this could go onto the conclusion, where it should be stressed that a certain risk was identified in this study and that now, further research is needed to explore the dimension of this risk, as well as certain measures to tackle it.

I have read this submission. I believe that I have an appropriate level of expertise to confirm that it is of an acceptable scientific standard, however I have significant reservations, as outlined above.

## References

[1] World Health Organization: Global status report on alcohol and health 2018.2018;

[2] HingsonRWHeerenTWinterMR: Age at drinking onset and alcohol dependence: age at onset, duration, and severity. *Arch Pediatr Adolesc Med*.2006;160(7) : 10.1001/archpedi.160.7.739 739-46 10.1001/archpedi.160.7.739 16818840

[3] GrantBFDawsonDA: Age at onset of alcohol use and its association with DSM-IV alcohol abuse and dependence: results from the National Longitudinal Alcohol Epidemiologic Survey. *J Subst Abuse*.1997;9:103-10 949494210.1016/s0899-3289(97)90009-2

# Peer review: Referee response for version 2

**DOI:** 10.5256/f1000research.15625.r32256

**Published:** 2018-03-22

**Authors:** Dirk W. Lachenmeier

**Affiliations:** 1 Chemisches und Veterinäruntersuchungsamt (CVUA) Karlsruhe, Karlsruhe, Germany

The authors have adequately considered all my previous comments.

 The paper could be further improved by an English language correction as some sentences are difficult to understand. For example:

 "However, there are traditional drinks that have alcohol content (Chicha de Jora-Clarito); artisanal drinks of traditional origin with alcoholic content in Peru."

 "Mothers provide accessibility to the beverages and perceive the risk the drinks have, which will more accurately evaluate this risk."

 Some further minor corrections:

 "thus reaching about 28.7% of unregistered alcohol": change unregistered to unrecorded (see WHO wording)

 "since consumers despite having between 10 to 12 degrees of alcohol, they consider this as a traditional drink". Change "degrees of alcohol" to "% volume". It is also illogical why the content of alcohol would exclude the status as "traditional drink".

I have read this submission. I believe that I have an appropriate level of expertise to confirm that it is of an acceptable scientific standard.

# Peer review: Referee response for version 1

**DOI:** 10.5256/f1000research.13025.r24709

**Published:** 2017-08-25

**Authors:** Paul Anthony Camacho López

**Affiliations:** 1 Research department, Fundación Oftalmológica de Santander FOSCAL, Bucaramanga, Colombia

The article is interesting, but the presented analysis only is descriptive, losing the relevance and applicability. I suggest that Authors enlarge the analysis, which should evaluate the potential risk factors. The conclusion is adequate, but only they could describe the consumption of traditional alcoholic beverages.

 The introduction is not enough to explain the problem compared to the use of alcoholic beverages. In the methods, authors should clarify the patterns of consumption, the frequencies of consumption per week or monthly. The data analysis only centered in the descriptive the mother´s consumption (perceptions), accessibility and perception of risk, but they did not analyze the association or relation with child´s age, weight, height and school grade, household income and number members.

I have read this submission. I believe that I have an appropriate level of expertise to confirm that it is of an acceptable scientific standard, however I have significant reservations, as outlined above.

# Response

**Published:** 2018-03-16

**Authors:** Martín Vilela

**Affiliations:** Universidad Privada Antenor Orrego, Peru

Dear reviewer, the requested changes have been made in the comments, in the same way, we communicate that this is a preliminary study and we take into account in a future study to consider data in associations, as well as associated factors.

 On behalf of the Authors, very grateful

# Peer review: Referee response for version 1

**DOI:** 10.5256/f1000research.13025.r24666

**Published:** 2017-07-31

**Authors:** Dirk W. Lachenmeier

**Affiliations:** 1 Chemisches und Veterinäruntersuchungsamt (CVUA) Karlsruhe, Karlsruhe, Germany

The authors provide a pilot study into the consumption of traditional alcoholic beverages in rural Peru. The article is interesting and novel as there is clearly a lack of data on unrecorded alcohol from South America. It is also quite disturbing to read that considerable alcohol exposure may occur in children.

 The following revisions should be considered:

1. For the international reader the beverages Chicha de Jora and Clarito are almost unknown. Can some information about these beverages be provided as background? For example, are they similar to maize beers? What is the typical alcoholic strength of these beverages? Are they commercially and legally sold by some kind of artisanal small-scale industry, or are they illegally sold? Should they be considered as falling into the WHO category of “unrecorded alcohol” (see Rehm et al. ^1^ for definition).
2. The conclusion states that the products are providing “great danger” to health. While this may be true, there is not much in the data that would allow for such a conclusion. The study appears to be non-quantitative in nature and the alcoholic strength of the product appears to be unknown. Hence no calculation of daily alcohol exposure can be made, which would allow for a quantitative risk assessment (such as by using the margin of exposure approach ^2^). With the currently available data I would suggest to conclude that there may be a health hazard, but quantitative intake assessment as well as chemical characterization of the beverages would be necessary for risk assessment.
3. The introduction of the abstract is written in a rather vague fashion. “... traditional drinks that may have alcohol content”. Are Ch and Cl without alcohol available? This should be clarified.
4. Introduction, last paragraph. The reference 9 mentions “Chicora de Jora”. Is this the same as “Chicha de Jora”? Reference 10 mentions “girl from Jora”? Are these translation mistakes? Are there any more reliable peer-reviewed references with background on the beverages than some theses? Can a link to the source of reference 10 be provided?
5. Methods, characteristics of drinking habits in liquids/beverages: what beverage is “gas”? Clarify that Chicha morada is non alcoholic.
6. Table 3: check values for line “dangerous”. Is this logical that both are “0”?
7. Discussion, first sentence: Please provide percentages to make the values more easy to compare with the data from Brazil and Colombia.
8. Page 6, first line: please clarify where the consumption has risen (in Peru?)
9. Page 6, 4 ^th^ line: “low cost of production”. Please provide some comparison for the low cost. Are the alcoholic beverages cheaper than non-alcoholic alternatives such as milk or fruit juices?
10. Page 6, 1 ^st^ paragraph, last line “no studies about consumption of alcohol and drugs by children”. I wonder about this request and why this is seen as unfortunate. I would find it highly unethical to study alcohol and drug consumption in children, and I can predict that we will never see such a study. This sentence should be deleted.
11. Discussion, last paragraph: please include the non-quantitative nature as limitation. Considering your comment that “the consumption of these traditional alcoholic beverages” is seen as “normal”, is there at least some information how much of the beverages is consumed by the children?
12. Conclusions: “it is concluded that children consume a large quantity of traditional alcoholic beverages”. This conclusion appears to be not founded in the data. No quantitative measurements were conducted.

I have read this submission. I believe that I have an appropriate level of expertise to confirm that it is of an acceptable scientific standard, however I have significant reservations, as outlined above.

## References

[1] RehmJKailasapillaiSLarsenERehmMXSamokhvalovAVShieldKDRoereckeMLachenmeierDW: A systematic review of the epidemiology of unrecorded alcohol consumption and the chemical composition of unrecorded alcohol. *Addiction*.2014;109(6) : 10.1111/add.12498 880-93 10.1111/add.12498 24467748

[2] LachenmeierDWKanteresFRehmJ: Epidemiology-based risk assessment using the benchmark dose/margin of exposure approach: the example of ethanol and liver cirrhosis. *Int J Epidemiol*.2011;40(1) : 10.1093/ije/dyq150 210-8 10.1093/ije/dyq150 20819784

# Response

**Published:** 2018-03-16

**Authors:** Martín Vilela

**Affiliations:** Universidad Privada Antenor Orrego, Peru

Dear reviewer, the changes requested in the comments have been made.

 On behalf of the Authors, very grateful
